# New insights into alternative splicing in rice using population-level transcriptomics

**DOI:** 10.1093/plcell/koae214

**Published:** 2024-07-23

**Authors:** Nicolas M Doll

**Affiliations:** Assistant Features Editor, The Plant Cell, American Society of Plant Biologists; Laboratoire Reproduction et Développement des Plantes, Univ Lyon, ENS de Lyon, UCB Lyon 1, CNRS, INRAE, F-69342 Lyon, France

In eukaryotes, several post-transcriptional modifications are required to produce mature mRNA from precursor mRNAs (pre-mRNA). One key modification is the splicing of introns. Within a cell or an organism, variation in pre-mRNA splicing such as the skipping of one or more exons, the use of alternative splicing sites, or the retention of an intron may occur. This process, known as alternative splicing (AS), increases gene expression diversity by allowing a single pre-mRNA to generate several distinct mRNA transcripts, each potentially encoding different proteins. AS is crucial throughout the plant life cycle and contributes to resistance against various stresses. In rice more than 50% of intron-containing genes undergo AS (Dong et al. 2018). AS requires a versatile and complex regulatory mechanism that enables the controlled production of different isoforms from unique pre-mRNAs depending on factors such as the tissue type or the exposure to a stress.

In recent work, **Hong Zhang and co-authors** conducted an extensive analysis of AS in the leaf of 250 rice accessions and the panicle of 193 accessions, encompassing both wild and domesticated rice (Zhang et al. 2024). AS was assessed through in-depth RNA-sequencing of the leaves and panicles in the different accessions. In the study, 50.4% and 43.7% of the genes expressed in the leaf and panicle, respectively, underwent AS, consistent with the percentage previously identified in roots (Dong et al. 2018). Tens of thousands of new isoforms were detected, increasing the number of annotated transcripts by around 20% in both tissues. Notably, the alternatively spliced genes are strongly different between the 2 tissues, with 43.7% of them being spliced specifically in either the leaf or the panicle.

**Figure 1.**
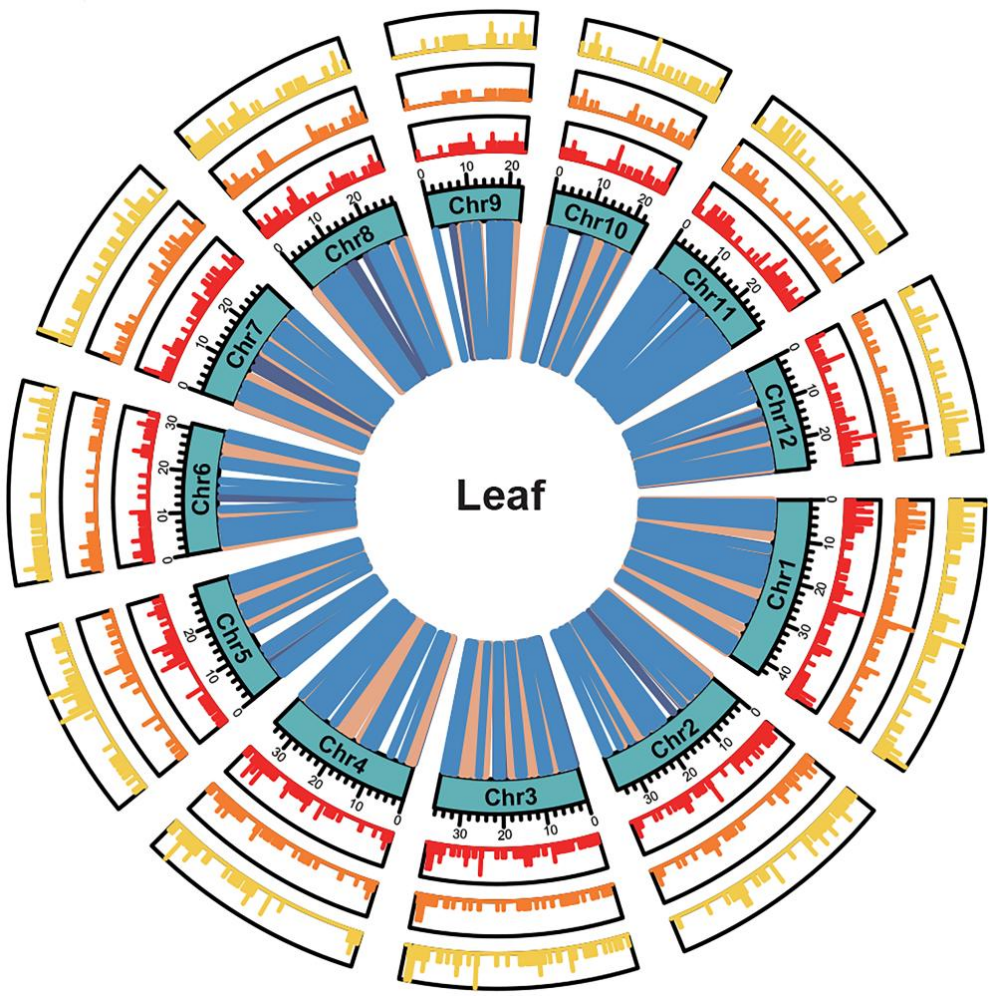
Distribution of the *cis*-sQTL identified in the leaf. The number of *cis*-sQTL SNPs (yellow, outer circle), *cis*-sQTL SVs (orange, middle circle), and spliced genes (red, inner circle) within a 100-kb window across the 12 rice chromosomes. Genes regulated by SNPs and SVs, SNPs alone, and SVs alone are represented by orange, blue, and purple lines, respectively. Reprinted from Zhang et al. (2024), Figure 3B.

To decipher the genetic regulation of AS, the authors performed a quantitative trait locus (QTL) analysis of the spliced genes (sQTL), using the relative percentage of spliced isoform as phenotype. They used the recently assembled super pan-genome of rice (Shang et al. 2022) to associate genetic variation to sQTLs. This analysis considered not only the single nucleotide polymorphism (SNPs) but also large structural genomic variation (SVs), whose impact on AS remains poorly understood. Both SNPs and SVs were shown to be associated with numerous sQTLs throughout the rice genome. These associations occurred either in *trans*, where the genetic variation is more than 40,000 base pairs away from the spliced gene, or in *cis* (Fig. 1).

*Cis*-sQTLs are mostly located in the regions flanking the splicing sites and the UTRs, highlighting the direct role of these regions in the splicing mechanism. In contrast, some *trans*-sQTLs correspond to a portion of the genome that contains genes encoding AS regulators. For example, the splicing factor *OsRH2* has a *trans*-sQTL associated with 12 spliced genes. The analysis of *trans*-sQTL provides interesting insights into potentially new regulators of splicing across the rice genome.

The data generated also allow the analysis of variation in AS between the main rice subpopulations. Notably, domesticated species and their wild ancestors produce several dozen different transcript isoforms in the leaf. This difference is also observed between *Oryza sativa japonica* and *Oryza sativa indica*, 2 domesticated species that had diverged after domestication. These findings indicate that AS has profoundly changed in the leaf throughout the domestication process and suggest that AS might be associated with agronomically favorable traits.

Consequently, the authors assessed the correlation between gene splicing ratios and 10 agronomic traits. Fifty-eight spliced genes in the leaf and 34 spliced genes in the panicle were associated with at least 1 agronomic trait. The strongest association was between plant height and *OsPIE1*, a gene encoding a phosphate starvation–induced RING-type E3 ligase. CRISPR/Cas9-mediated knockout of *OsPIE1* resulted in significantly smaller plants, validating the approach of using AS variation to identify new agronomically favorable genes.

In conclusion, this study provides an extensive description of AS in rice, giving numerous insights into AS regulation. It highlights the importance of AS in regulating phenotypic variation in rice and underscores its potential for future rice breeding efforts.
